# Molecular Epidemiology and Characterization of Picobirnavirus in Wild Deer and Cattle from Australia: Evidence of Genogroup I and II in the Upper Respiratory Tract

**DOI:** 10.3390/v13081492

**Published:** 2021-07-29

**Authors:** Jose L. Huaman, Carlo Pacioni, Subir Sarker, Mark Doyle, David M. Forsyth, Anthony Pople, Jordan O. Hampton, Teresa G. Carvalho, Karla J. Helbig

**Affiliations:** 1 Department of Physiology, Anatomy and Microbiology, School of Life Sciences, La Trobe University, Melbourne, VIC 3086, Australia; j.huamantorres@latrobe.edu.au (J.L.H.); s.sarker@latrobe.edu.au (S.S.); t.carvalho@latrobe.edu.au (T.G.C.); 2 Department of Environment, Land, Water and Planning, Arthur Rylah Institute for Environmental Research, Heidelberg, VIC 3084, Australia; carlo.pacioni@delwp.vic.gov.au; 3 Environmental and Conservation Sciences, Murdoch University, South Street, Murdoch, WA 6150, Australia; 4 South East Local Land Services, Bega, NSW 2550, Australia; mark.doyle@lls.nsw.gov.au; 5 Vertebrate Pest Research Unit, Department of Primary Industries, Orange Agricultural Institute, Orange, NSW 2800, Australia; dave.fosyth@dpi.nsw.gov.au; 6 Department of Agriculture and Fisheries, Invasive Plants & Animals Research, Biosecurity Queensland, Ecosciences Precinct, Brisbane, QLD 4102, Australia; tony.pople@daf.qld.gov.au; 7 School of Veterinary and Life Sciences, Murdoch University, South Street, Murdoch, WA 6150, Australia; jordan.hampton@unimelb.edu.au; 8 Ecotone Wildlife, Inverloch, VIC 3996, Australia; 9 Faculty of Veterinary and Agricultural Sciences, University of Melbourne, Parkville, VIC 3052, Australia

**Keywords:** cattle, deer, genetic diversity, metagenomics, picobirnavirus, RNA-dependent RNA polymerase

## Abstract

Picobirnaviruses (PBVs) have been detected in several species of animals worldwide; however, data pertaining to their presence in Australian wild and domestic animals are limited. Although PBVs are mostly found in faecal samples, their detection in blood and respiratory tract samples raises questions concerning their tropism and pathogenicity. We report here PBV detection in wild deer and cattle from southeastern Australia. Through metagenomics, the presence of PBV genogroups I (GI) and II (GII) were detected in deer serum and plasma. Molecular epidemiology studies targeting the partial RNA-dependent RNA polymerase gene were performed in a wide range of specimens (serum, faeces, spleen, lung, nasal swabs, and trachea) collected from wild deer and cattle, with PCR amplification obtained in all specimen types except lung and spleen. Our results reveal the predominance of GI and concomitant detection of both genogroups in wild deer and cattle. In concordance with other studies, the detected GI sequences displayed high genetic diversity, however in contrast, GII sequences clustered into three distinct clades. Detection of both genogroups in the upper respiratory tract (trachea and nasal swab) of deer in the present study gives more evidence about the respiratory tract tropism of PBV. Although much remains unknown about the epidemiology and tropism of PBVs, our study suggests a wide distribution of these viruses in southeastern Australia.

## 1. Introduction

Picobirnaviruses (PBVs) are members of the family Picobirnaviridae and have a non-enveloped bi-segmented double-stranded RNA (dsRNA) genome measuring 35–40 nm in diameter. The genome of a PBV consists of two segments: segment 1 (~2.4–2.7 kb in size) containing the capsid gene and an additional open reading frame, which encodes a putative protein of an unknown function; and segment 2 (~1.7–1.9 kb in size), containing the RNA-dependent RNA polymerase (RdRp) gene [1,2]. Based on the genetic variability of genomic segment 2, PBVs are classified into three genogroups [1]. Genogroup I (GI) has a wider distribution and usually a much higher prevalence than GII, which is reflected in the GenBank database, where over 80% of PBV sequences are GI [2]. Conversely, GIII has only been obtained from invertebrate hosts and differs largely from the PBVs of GI and GII [1].

PBVs were initially discovered in a small number of faecal specimens during the investigation of gastroenteritis in children in 1988 [3]. Since then, PBVs have been reported in humans, invertebrates, environmental water samples, and a wide range of vertebrates worldwide [1,2,4,5]. However, there is only one published report of PBV detection in cervid (deer) species. In this study, 60% (42/70) of faeces collected from roe deer (*Capreolus capreolus*) in Slovenia tested positive for PBV GI by polymerase chain reaction (PCR) [6]. In Australia, PBVs have been previously detected by metagenomics in stool samples collected from humans [4], wild birds [7,8], European rabbits (*Oryctolagus cuniculus*) [9], and Tasmanian devils (*Sarcophilus harrisii*) [10].

Although PBVs have been detected in faeces/gut contents of humans and various animal species with diarrhoea, the role of PBV as a causative agent of diarrhoea is still controversial. Indeed, its presence has also been reported in asymptomatic hosts, leading to the assumption that PBVs may be opportunistic enteric pathogens [1,2,4,11]. However, PBVs have also been detected in the respiratory tract of humans [12,13], domestic pigs [14], cattle and monkeys (*Macaca mulatta* and *Macaca fascicularis*) [15], and in the plasma of cattle [16] and domestic horses [17], suggesting an expanded tissue tropism of the virus.

To date, the difference in frequency and distribution between GI and GII is considerable, with a higher prevalence of GI [14,15,18,19] when both genogroups can be detected in a given population and the detection of only GI in some studies [11,20,21,22,23,24,25,26]. However, the presence of both genogroups has also been seen in respiratory tract samples [14,15]. Phylogenetic analysis of PBV GI reveals high genetic diversity between and within host species, and species-specific clustering patterns have not been observed so far [6,19,20,27]. Interspecies transmission events, including zoonoses, have been proposed for PBV GI [4,5,11,19,26], based on sequence identities and phylogenetic analysis; however, the use of partial gene segment-2 sequences in several of these studies make these findings inconclusive. Moreover, until the true host/s of PBVs are proven, caution should be exercised when interpreting PBV interspecies transmission events [28].

The aims of the present study were: (i) to detect novel RNA viruses in serum and plasma samples collected in wild deer from southeastern Australia, and (ii) to expand our knowledge on the epidemiology of PBV by investigating their presence and quantifying their genetic diversity in wild deer and cattle from southeastern Australia. These objectives were achieved by high-throughput sequencing and RT-PCR coupled with Sanger sequencing. This study reports for the first time the presence, molecular characterisation, and genotypic differentiation of PBVs in a wide range of samples from wild deer and cattle.

## 2. Materials and Methods

### 2.1. High-Throughput Sequencing

#### 2.1.1. Samples

**Figure 1:**
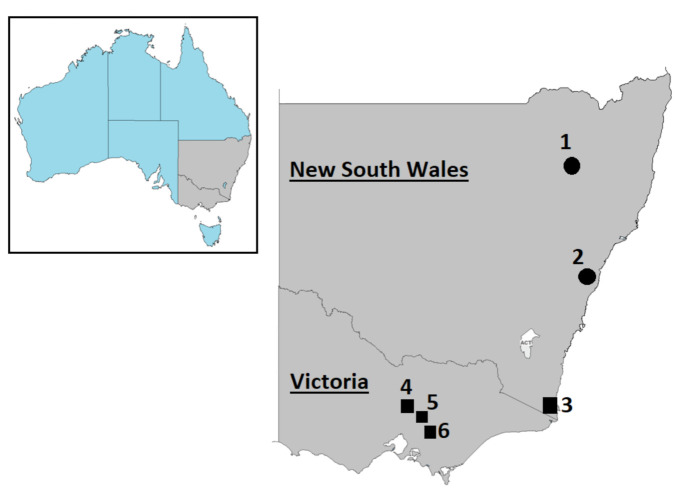
Geographic location of deer (1–6) and cattle (3, 4, and 6) sample collection in southeastern Australia. ● indicates sampling sites of serum and plasma tested by high-throughput sequencing, while ■ indicates sampling sites of specimens tested by PCR. (1) Liverpool Plains, (2) Wollongong, (3) Kiah, (4) Outer Melbourne, (5) Yellingbo, (6) Bunyip. ©d-maps.com (25 June 2021).

This study used samples collected as part of a disease investigation in Australian wild deer [29]. Briefly, blood samples were collected during culling of fallow deer (*Dama dama*) in the Liverpool Plains and rusa deer (*Rusa timorensis*) at Wollongong, New South Wales (Figure 1). Blood was drawn from the jugular vein and/or the heart and thoracic cavity and collected in tubes (Becton Dickinson, Franklin Lakes, NJ, USA) with and without anticoagulant (EDTA) to obtain plasma and serum, respectively. Serum and plasma were separated by centrifugation (10 min at 2000× *g*). Three serum and two plasma samples were selected for this analysis. All samples were stored at −80 °C until next-generation sequencing was carried out.

#### 2.1.2. Virion Enrichment and Virus Nucleic Acid Extraction

In order to remove impurities such as host cells and bacteria, viral enrichment was conducted as per published protocols with minor modifications [30,31]. Briefly, samples were filtered through 0.8 μm polyethersulfone (PES) (Sartorius cat#: VK01P042) spin filters at 17,000× *g* for 1 min. Filtrates were then nuclease treated using benzonase and micrococcal nuclease and incubated at 37 °C for 2 h, followed by nucleic acid extraction using the QIAamp Viral RNA mini kit (Qiagen, Valencia, CA, USA) without adding any carrier RNA. This approach enabled both DNA and RNA virus nucleic acids to be extracted using this kit.

#### 2.1.3. Random Amplification

cDNA synthesis and amplification were performed using the Whole Transcriptome Amplification Kit (WTA2, Sigma-Aldrich, Darmstadt, Germany). WTA2 PCR products were then purified using the Wizard^®^ SV Gel and PCR Clean-Up kit (Promega, Madison, WI, USA) and quantified using a Qubit dsDNA high sensitivity assay kit with Qubit Fluorometer v3.0 (Thermo Fisher Scientific, Waltham, MA, USA).

#### 2.1.4. Library Preparation and Illumina Sequencing

Sequencing libraries were constructed using the Nextera XT Flexi DNA Library kit (Illumina, San Diego, CA, USA) according to the manufacturer’s instructions. Libraries were then pooled and sequenced with the Illumina Hiseq2500 platform and using a 2 × 150 PE high-output flow cell at the Australian Genome Research Facility, Melbourne, Australia.

#### 2.1.5. Bioinformatic Analysis

The raw data were demultiplexed, and Trim_Galore v0.4.5 was used for quality control of the reads, with the minimum sequence length set to 50 bp and Phred Quality of 25. Host genome reads were filtered out by mapping the reads to deer RefSeq genome (GenBank GCF_002102435.1) using bwa v0.7.17, samtools v1.6, and bedtools v2.26. The cleaned reads were de novo assembled using SPAdes v3.10.1 [32] with parameters-careful-cov-cutoff-k 21, 33, 55, 77, 99, 127. The resulting contigs were compared against the nonredundant nucleotide and protein databases on GenBank using BLASTn and BLASTx, respectively, with an e-value threshold of 1 × 10^−5^ to remove potential false positives. All contigs that returned blast hits were filtered to remove non-viral reads that likely correspond to fungal or bacterial species. Virus contigs of interest (>500 nucleotides and query cover >50%) were imported in Geneious software (Biomatters Ltd., Auckland, New Zealand, version 11.1.4), and putative ORFs were predicted using the same software. Multiple alignment with reference sequences was performed using MAFFT (version 7) [33]. Phylogenetic trees of aligned amino acid or nucleotide sequences were then inferred using the maximum-likelihood method implemented in MEGA (version 7) [34], using the best-fit substitution model based on the lowest BIC scores as determined by jModelTest. Statistical support for the trees was evaluated by bootstrapping based on 1000 repetitions.

### 2.2. PBV PCR Detection and Sanger Sequencing

#### 2.2.1. Sampling

Deer culled for other reasons were opportunistically sampled during field necropsies with the assistance of recreational and professional hunters during August 2019 and October 2020 in New South Wales and Victoria (Figure 1). Sampling sites were Yellingbo Natural Conservation Reserve and Bunyip State Park; however, for the purposes of this study, we used Yellingbo and Bunyip to denote sampling in these areas. To the best of our knowledge, the deer sampled in our study showed no apparent evidence of disease. Blood was drawn from the jugular vein and/or the heart and thoracic cavity and collected in tubes (Becton Dickinson, Franklin Lakes, NJ, USA) without anticoagulant (EDTA). Serum was separated by centrifugation (10 min at 2000× *g*). Lung, trachea, and spleen samples were collected from each carcass and treated with homemade RNA and DNA preservation solution consisting of 7.44 g of EDTA, 7.35 g of sodium citrate trisodium salt dihydrate, and 700 g of ammonium sulfate in 1 L of water [35]. Nasal swabs were taken with sterile swabs and kept in viral transport medium (Eagle Minimum Essential Medium, 0.5% bovine serum albumin, gentamicin 250 mg/L, streptomycin 200 mg/L, and amphotericin B 0.5 mg/L). Deer faecal specimens were collected from the large intestine and individually placed in sterile plastic containers. Additionally, opportunistic cattle faecal samples collected for other purposes (i.e., clinical purposes) from farms within 20 km of deer sampling areas were included in the present study. All sample containers and tubes were immediately refrigerated after collection, then transported to the Laboratory of Virology within the Department of Physiology, Anatomy and Microbiology at La Trobe University, and stored at −80 °C until further analysis.

#### 2.2.2. RNA Extraction

Aliquots of 100 mg (0.1 g) of spleen, lung, and trachea specimens were used for RNA extraction using 1 mL TRIsure™ (Bioline, London, UK), and following the manufacturer’s instructions. After drying, the RNA pellet was resuspended in 30 μL of DEPC-treated water and used as the template for cDNA synthesis. Faecal specimens were converted to 10% (*w*/*v*) suspensions in phosphate-buffered saline (PBS pH 7.2) for RNA extraction. A total of 0.1 g of thawed sample was suspended in 1 mL of PBS. The suspension was stored overnight at −80 °C, then centrifuged at 4000× *g* for 15 min at 4 °C. RNA was extracted from serum, transport media (nasal swab), and 10% faecal suspensions using a QIAamp^®^ Viral RNA Mini Kit (Qiagen, Valencia, CA, USA), according to the manufacturer’s instructions. The RNA was eluted in 50 µL of resuspension buffer and used as the template for cDNA synthesis.

#### 2.2.3. cDNA Synthesis, PCR Amplification, and Sequencing

The viral RNA was reverse transcribed using Tetro cDNA Synthesis Kit (Bioline, London, UK) with random hexamers, following the manufacturer’s instructions. The cDNA was used as a template for the PCRs to detect the two PBV genogroups (I, II). All primers amplified PCR product fragments of different sizes of segment 2’s RdRp gene. To detect genogroup I, primers AACCCAAATTCACAGTGTCTTGG and AGAGGATGGTACTTCACATTCTC [6] were used to amplify a PCR product of 326 bp. Primers WTGGATGTTTCCGATGTC and TGYGCATCCATYTTMGTGGTGTCTC [15] were used to detect genogroup II by amplifying a PCR product of 207 bp. PCR amplification was performed in a 25 μL reaction mixture containing 1× Green GoTaq Flexi buffer, 2 mM of MgCl2, 10 mM of dNTPs, 0.2 μM of both forward and reverse primers, 0.625 units of GoTaq G2 DNA polymerase (Promega, Madison, WI, USA), and 1 μL of cDNA. Amplification was conducted in a T100 thermal cycler (BioRad, Hercules, CA, USA), and PCR products visualised by gel electrophoresis, using 2% agarose gel, RedSafe™ (iNtRON Biotechnology, Gyeonggi-do, Korea), and a high-resolution imaging system (ChemiDoc™ MP Imaging System (BioRad, Hercules, CA, USA)). All PCR products were purified using the Wizard^®^ SV Gel and PCR Clean-Up kit (Promega, Madison, WI, USA) according to the manufacturer’s instructions. Nucleotide sequencing was performed by Sanger sequencing at the Australian Genome Research Facility, Melbourne, Australia. Sequences were deposited in GenBank under accession numbers MZ048540-MZ048585 (genogroup I) and MZ048586-MZ048608 (genogroup II).

#### 2.2.4. Precautions to Avoid Contamination

To avoid contamination of our results, we took a number of precautions, including the use of a laminar flow cabinet equipped with a UV lamp for aliquoting and preparing samples. Additionally, RNA extraction, PCR preparation, and PCR were performed in physically separated rooms, and filter pipette tips were used for all pipetting. Extraction of RNA-negative samples (nuclease-free water) was also included for each extraction round and as negative controls in every PCR run.

### 2.3. Phylogenetic Analysis of GI and GII

Nucleotide sequences were edited using Geneious software (Biomatters Ltd., Auckland, New Zealand, version 11.1.4), and the final sequences were compared with PBV sequences deposited in the NCBI GenBank database, using the BLAST server [36]. Some GI nucleotide sequences investigated in this study were trimmed to 243 bp due to low-quality bases at both ends. BLASTn searching confirmed the similarity of the sequences obtained with GI or GII isolates, so the cognate PBVs from GenBank were included in the phylogenetic analysis. The PBV strains 1-CHN-97 (AF246939) and 4-GA-91 (AF246940) belonging to GI and GII, respectively, were used as representative strains and as outgroup sequences [1]. Nucleotide sequences were aligned using the MAFFT program (version 7), and identities were determined from the same alignment using Geneious software. The best-fitting nucleotide substitution model (T92 (Tamura 3-parameter) +G+I for GI and GTR (general time-reversible) +G+I for GII) was determined based on the lowest BIC scores in MEGA 7 [34]. Phylogenetic trees were also constructed with this software, using the maximum-likelihood method. Statistical support for the trees was evaluated by bootstrapping based on 1000 repetitions.

### 2.4. Phylodynamic Analysis of GII

The geographic dynamics of the PBV GII detected in the present study were analysed under the structured coalescent model [37] in BEAST v2.6.3 [38] using the MultiTypeTree package [37]. The southern and northern sampling sites were codified as Victoria and New South Wales. The general time-reversible (GTR) nucleotide substitution model was used with a relaxed lognormal clock. Two independent runs of 50 million steps were computed, sampling parameters every 10,000 steps and discarding 10% of each chain as burn-in. Tracer v1.7.1 [39] was used to ensure that the length of the burn-in phase was sufficient and check the convergence of the two analyses. Results were obtained after combining the two chains with LogCombiner. The programs TreeAnnotator v2.6.2 and FigTree v1.4.4 were used to summarise the posterior tree distribution and to visualise the annotated maximum clade credibility (MCC) tree, respectively.

## 3. Results

### 3.1. High-Throughput Sequencing

Illumina sequencing of five deer sample libraries was initially performed to investigate the presence of novel RNA viruses in a sub-section of deer serum samples. Sequencing generated a total of 205,614,103 paired-end (PE) reads with library size ranges of 38,934,907 to 47,809,012 PE reads. After trimming, 93% of the reads (191 million) were retained, with 60% of these retained trimmed reads (115 million) not mapping to published host DNA. In each of the libraries, phages made up at least 80% of the total number of viral contigs. Further analysis of eukaryotic viral contigs revealed sequences identified as picobirnaviruses segment 2 (or RdRp) in four deer samples with lengths ranging from 881 to 1719 nucleotides (Table 1).

**Figure 2:**
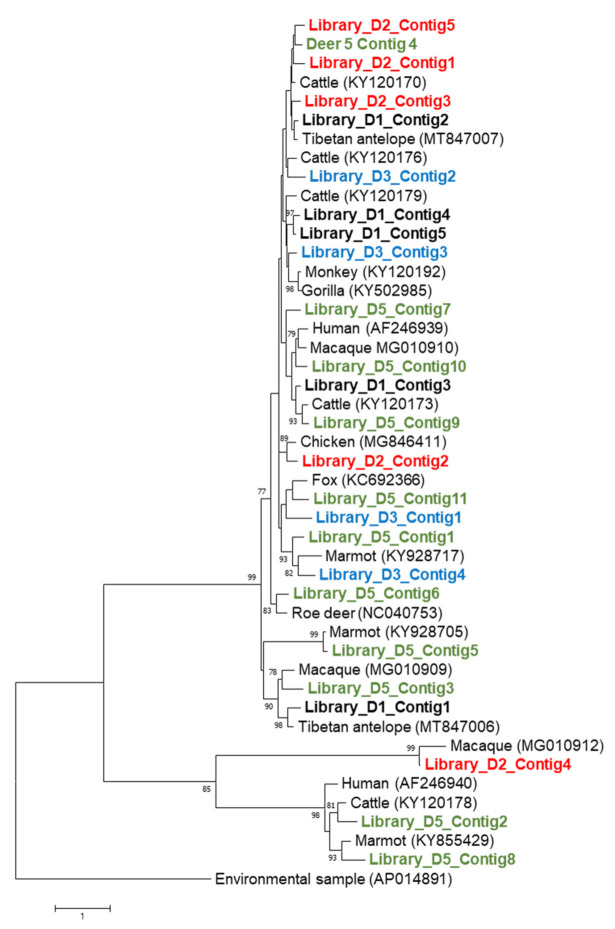
Phylogenetic analysis of translated sequences matched with the RdRp gene of PBVs discovered by high-throughput sequencing in the present study. The tree was constructed using the maximum-likelihood method and the optimal substitution model of LG+G. The tree was unrooted and included sequences of the three genogroups. Bootstrap values below 70% are not shown. Contigs obtained from the same animal are highlighted with the same colour.

We also searched for protein-coding regions in the detected contigs and found that the predicted ORFs were consistent with RdRp fragments that have been reported previously in PBV detected in humans and other animal species [1,4]. Thus, several PBV clones were identified in deer serum and plasma samples with contigs showing amino acid (aa) identities from 59% to 87% with PBV sequences already deposited in the GenBank database (Table 1). To determine genetic relationships between aa-translated contigs with their cognate PBVs, a phylogenetic tree was constructed based on the RdRP gene. Representative strains of GI (Human/AF246939), GII (Human/AF246940), and GIII (Environmental sample/AP014891) [1] were also included in the tree. Phylogenetic analysis of amino acid translated contigs (Figure 2) showed: (i) viruses grouped, with substantial support, with members of GI or GII; (ii) GI sequences were found in all samples; (iii) GI sequences had a high genetic diversity, with more than one clone in each library, being distributed throughout GI among picobirnaviruses from different hosts without forming a monophyletic group; and (iv) GII sequences were detected in library_D2 and library_D5. Although most of the PBV detected in the same deer library did not cluster together, contig 4 and contig 5 from library_D1 formed a highly supported clade with a bootstrap of 97%, and nt and aa identity of 86% and 87%, respectively. In addition, contigs from library_D1 and library_D5 formed highly supported clades with a bootstrap >90% and aa identities of 68% to 86% (Table 1) with PBVs detected in cattle, marmot (*Marmota himalayana*), and Tibetan antelope (*Pantholops hodgsonii*) (Figure 2).

### 3.2. PBV PCR Detection and Sanger Sequencing

In order to investigate the prevalence of PBVs in deer and cattle, different tissues (serum, faeces, spleen, lung, nasal swab, and trachea) were tested by RT-PCR. Serum and faeces samples were collected from 71 deer (Table 2), including 48, 5, and 7 fallow deer sampled in Kiah, Yellingbo, and Bunyip, respectively. The remaining 11 deer, all sambar deer (*Rusa unicolor*), were sampled in Outer Melbourne, Yellingbo, and Bunyip (Figure 1). From 15 fallow deer sampled in Kiah, spleen, lung, trachea, nasal swabs were also collected (Table 2). Of the 71 deer sampled, 36 (50.7%) tested positive for PBV, with the majority of those being identified as GI PBV, which is consistent with previous studies [14,15,18,19]. In addition, evidence of both genogroups was found in 22.2% of deer positive for PBV. Picobirnavirus was detected in all tissues collected except spleen and lung, with nasal swabs and faeces showing the highest positive detection rate. (Table 2). A total of 19 out of 23 (82.6%) of the cattle samples tested positive for PBV by PCR, and as was observed for the deer samples, a high GI prevalence was obtained from cattle samples. Furthermore, detection of both GI and GII was found in 36.8% of cattle with PBV-positive samples (Table 3).

There were 36 deer samples that tested positive for either PBV GI (*n* = 7) or GII (*n* = 3), sourced across multiple specimen types. For PBV genogroup I, the most frequent multi-specimen detection was faeces/serum, with one deer testing positive in three different specimens (Table 4). Conversely, faeces/nasal swab was mostly detected for GII, and two deer tested positive in three different specimens (Table 5). Interestingly, the trachea and nasal swab samples from the deer labelled N329 tested positive for both GI and GII (Table 4 and Table 5).

### 3.3. Phylogenetic Analysis of PBV GI Samples

To determine genetic relationships between deer PBV GI samples and cognate PBVs (from other animals and humans), a phylogenetic tree was constructed based on a partial fragment of the RdRP gene, as has been performed previously [6]. Nucleotide sequences were obtained from all 56 PCR-positive products, but due to the low quality observed in 4 sequences, only 52 were used for further phylogenetic analysis. In total, 74 nucleotide sequences were aligned, including 37 from a range of deer sampling locations, 15 from cattle faecal samples, and 22 selected sequences of PBV strains from domestic pigs, chickens, camels, sea lions, cattle, Tibetan antelope, monkeys (*Macaca* spp.), and humans derived from GenBank. The 4 GA-91 strain (AF246940) was used in the phylogenetic analysis as an outgroup.

**Figure 3:**
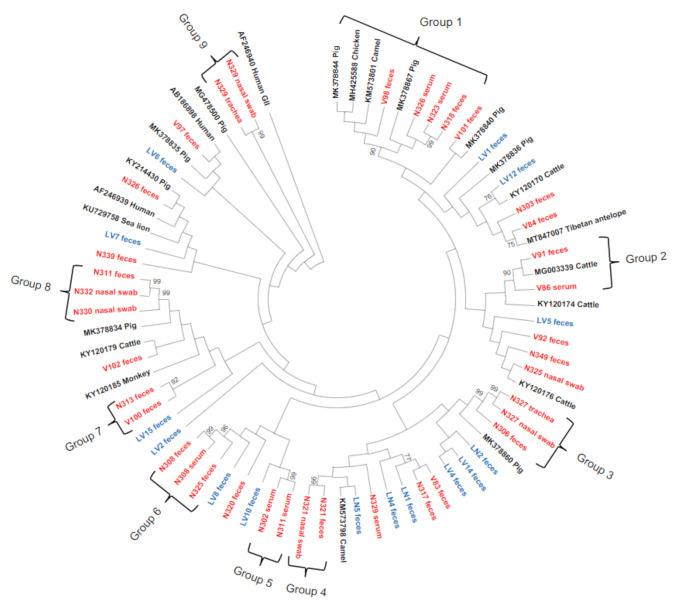
Phylogenetic analysis of partial RdRp gene of PBVs GI discovered in the present study. The tree was constructed using the maximum-likelihood method and the optimal substitution model of T92+G+I and rooted with genogroup II human strain AF246940. Two hundred and 43 nucleotide positions were included in the analysis. Bootstrap values below 70% are not shown. Deer sequences are highlighted in red and cattle sequences in blue.

The phylogenetic tree (Figure 3) revealed that deer and cattle strains were highly divergent, with pairwise comparisons between deer and cattle sequences showing nucleotide identities ranging from 64.9% to 100%. Moreover, nine highly supported groups (with bootstrap values ≥90% and nucleotide identity ≥80%) of deer PBV sequences were observed on the phylogenetic tree, with two of these groups including PBVs from other animals. Nucleotide identities within the nine groups ranged from 88.8% to 100%.

The high nucleotide identities obtained in some groups suggest the presence of the same PBV clone across samples. Although nucleotide identities >95% and a high bootstrap value of 99% were observed in most of the PBVs detected in different specimens sourced from the same animal (Table 4), the distribution in distinct lineages and the low nucleotide identity shared by N311 (faeces and nasal swab—80.3%), N325 (faeces and nasal swabs—80.8%), N326 (faeces and nasal swab—79.3%), and N329 (serum and trachea or nasal swab—70.8%) may indicate the presence of different PBV clones in these specimens. Phylogenetic analysis of the cattle PBV samples showed distribution over the whole phylogenetic tree, but the highest similarity between these samples was found among LV4 and LV14 with 92.9%. Moreover, the closest related sequence was bovine PBV (KY120170), sharing 90.4% of the sequence with LV12.

### 3.4. Phylogenetic and Phylodynamic Analysis of PBV GII Samples

**Figure 4:**
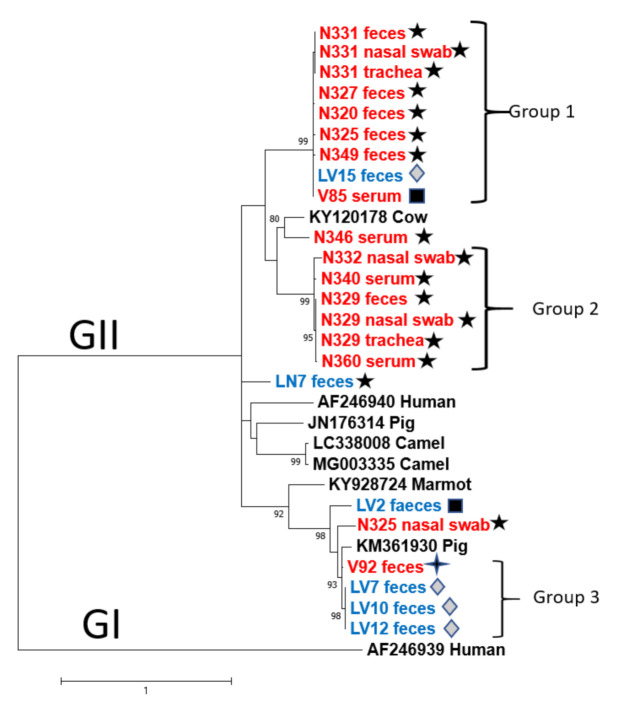
Phylogenetic analysis of partial RdRp gene of PBVs GII discovered in the present study. The tree was constructed using the maximum-likelihood method and the optimal substitution model of GTR+G+I and rooted with GII human strain AF246940. One hundred and ninety-three nucleotide positions were included in the analysis. Bootstrap values below 70% are not shown. Deer sequences are highlighted in red and cattle sequences in blue. ★ indicates samples collected in Kiah—NSW, 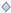 samples collected in Bunyip—VIC, ◼ samples collected in Outer Melbourne—VIC, and 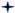 samples collected in Yellingbo—VIC.

The animal PBV sequence most closely related to both deer and cattle PBVs was the porcine PBV (KM361930) with 93.7% nucleotide identity compared to V92 faeces and 92% with LV7, LV10, and LV12. To determine genetic relationships between deer PBV GII sequences with cognate PBVs, a phylogenetic tree was constructed based on a 193 bp fragment of the RdRP gene, as has been performed previously [15] (Figure 4). In total, 32 nucleotide sequences were included in the tree, 17 from deer across multiple sampling sites, 6 from cattle faecal samples, and 7 selected sequences derived from GenBank, including the representative GII strain 4-GA-91 (AF246940) [1]. The 1-CHN-97 strain (AF246939) was used in the phylogenetic analysis as an outgroup. Overall nucleotide identity between the 17 deer sequences ranged from 57% to 100%, with the 6 cattle PBV sequences showing nucleotide identities from 60.6% to 100%.

Phylogenetic analysis demonstrated that the GII samples were distributed between eight separate branches, with three highly supported groups (with bootstrap values ≥90% and nucleotide identity >80%), suggesting relationships between the GII circulating in these areas (Figure 4). High nucleotide identities among the three groups suggest that these animals might be infected with the same PBV clone, but their geographical distributions were variable among divergent groups. The geographical distribution of all animals in the second group was restricted to New South Wales only, however within the first group, one cow and one deer sequence from Victorian regions clustered with New South Wales deer PBV sequences. In addition, cow LN7 and deer N346 serum clustered in different branches and were distant from other cow or deer samples (Figure 4).

Identical sequences and monophyletic clustering were obtained in PBV GII sourced from different specimens in deer N329 and N331 (Table 5 and Figure 4). The distribution in distinct lineages and the low nucleotide identity (61%) shared by faeces and nasal swab samples collected from deer N325 indicates the presence of different PBV clones in this animal. Phylogenetic analysis of the cattle PBV sequences showed that they were distributed in four different branches. One of these lineages clustered samples LV7, LV10, and LV12, which presented the highest nucleotide identity among cattle PBV GII sequences (100%). Interestingly, nucleotide alignment of all PBV GII sequences obtained in this study revealed a three-nucleotide deletion in position 144–146 (samples clustered in the second group, LN7 faeces and N346 serum), and a single nucleotide deletion in position 40 (samples clustered in the third group, LV2 faeces and N325 nasal swab) (see Figure S1).

**Figure 5:**
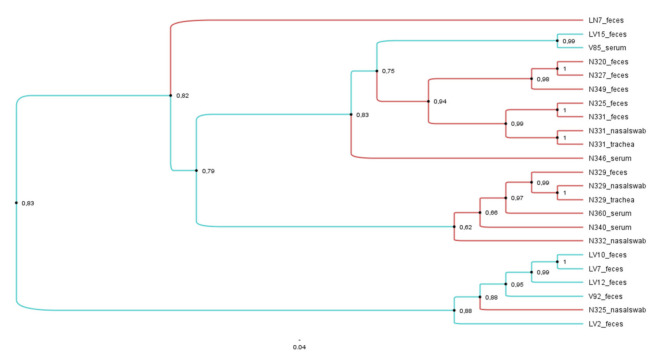
Spatial dynamics of Australian PBV GII. Maximum clade credibility phylogeny inferred under a Bayesian structured coalescent model. The nodes are annotated with the location posterior probabilities. Branches are blue for Victoria and red for New South Wales.

We next constructed a maximum clade credibility tree using sequences of PBV GII detected in deer and cattle to understand the transmission dynamics in southeastern Australia between these two species. This tree showed a similar topology to the previously constructed phylogenetic tree (Figure 4). The Victoria median population size estimated by the structured coalescent analysis was approximately eight times higher than NSW, although HPD intervals were overlapping (NSW median: 0.02, 95% highest probability density—HPD—0.0002–0.18; VIC median: 0.24, 95% HPD 4.2 × 10^−5^–0.49). Migration rate from NSW to VIC was median 71.77, 95% HPD 7.6 × 10^−7^–613.34) and from VIC to NSW was median 0.93 (95% HPD 2.21 × 10^−7^–48.36). The location of most recent common ancestor of the PBV GII detected in the present study was in Victoria, with a relatively high posterior probability (83%) (Figure 5).

## 4. Discussion

Australia’s wild deer populations have increased in abundance and distribution during recent decades [40], and the close interactions between deer and livestock are a risk for pathogen transmission [41]. However, little is known about the epidemiology of pathogens that wild deer in Australia may transmit to livestock, other domestic animals, or wildlife. A thorough understanding of the diversity of viruses, their transmission routes, and their tropism in wild deer provides epidemiologic baseline information about potential pathogenic threats for humans, domestic animals, and other wildlife species. The present study complements our initial work about the viral investigation in wild deer across multiple geographic locations in Australia [29]. Picobirnaviruses were detected via deep sequencing analysis of a small group of deer serum and plasma samples, with subsequent molecular screening being performed in a range of specimens collected from wild deer in southeastern Australia, as well as in faecal samples from cattle. This baseline information is of value for monitoring the status of viral infections in Australian deer and for evaluating the risk of disease transmission between wild deer and livestock. Additionally, it increases our knowledge of the potential transmission dynamics of PBVs, a group of viruses for which we have a limited understanding to date.

To our knowledge, this study reports for the first time: (i) the presence, molecular characterisation, and phylogenetic relationship of PBVs infecting deer and cattle in Australia, and (ii) the screening of PBVs in a wide range of sample types (faeces, serum, spleen, trachea, lung, and nasal swab) from the same animal. To date, only two reports have detected PBV in blood [16,17] by high-throughput sequencing. Wang et al. [16] found three reads that matched dromedary PBV in bovine plasma, and Li et al. [17] reported four different and not phylogenetically related PBV segment 2 (RdRp) contigs in a pool of plasma sourced from six diseased horses. Four out of five samples analysed by high-throughput sequencing in the present study showed contigs that matched PBVs. Furthermore, analysis of viral RdRp sequences revealed that at least four highly distinct picobirnaviruses in each animal. The presence of multiple PBVs has been previously reported in studies exploring viromes in stool specimens [9,10] and plasma [17]. Surprisingly, the library_D5 showed sequences for nine different GI clones. This high number of PBV contigs per sample is perhaps not surprising, given the extreme sequence diversity displayed by genogroup 1 PBVs in mammals [19,27,28]. However, there is also a possibility that this finding might be due to contamination of the blood with faeces strains. Samples selected for high-throughput sequencing were collected during aerial culling operations where the shot can occasionally result in perforation of the intestines or stomach, which could contaminate the bloodstream. However, this possible cross-contamination seems to be rare in the PCR screening. Here, samples were collected during ground culling operations, where animals are mostly shot in the head. In fact, our PCR screening results showed distinct strains in serum and faeces. From the 71 deer that both blood and faeces were collected and screened by PCR, serum/faeces-positive amplification was found only for PBV GI, with identical sequences found in one of them (N308). Given the extreme sequence diversity within the PBV GI, which has been reported previously [19,27,28], a more complete analysis of this genogroup might be found if future studies were to use a more long-read sequencing platform such as the Oxford nanopore technology [42].

Our findings describe the existence of PBV GI and GII in deer and livestock in Australia. Both genogroups were detected in the same animal, suggesting that they can co-infect one single host simultaneously. Notably, similar to other reports in the literature [2,4], PBV GI were more commonly found than GII, and the phylogenetic tree analysis of their sequences confirmed the high levels of genetic diversity among PBVs GI in our samples. These results corroborate previous studies that demonstrated the high genetic diversity of PBV GI observed worldwide, with no subtypes related to host species or geographic location in general [15,18,19,20,27]. Furthermore, it has been reported that the RdRp-coding genes of dsRNA and some ssRNA viruses vary outside the conserved motif regions, explaining the diversity observed between different members of the same genogroup [43]. Conversely, PBV GII isolates displayed a conservative pattern with high genetic similarity forming three clades, with two of them including cattle isolates. Similar observations were found regarding the high genetic diversity of porcine respiratory genogroup I picobirnaviruses [19], and cross-species transmission of picobirnaviruses between porcine and humans has been suggested based on the genetic relationships of their strains [19,44]. Similarly, in this study, the high nucleotide identity found in deer and cattle PBV GII samples sourced from the same geographical area (Victoria) suggests the presence of the same PBV and a possible transmission between these species.

No amplification of PBV sequences was detected in the lung or spleen, suggesting the absence of chronic systemic infection in the wild deer sampled for this study. Since PBVs have been mostly detected in faeces/gut contents of animals and humans with or without diarrhoea, they were considered as opportunistic enteric pathogens of mammals [4,28]. In fact, the detection of identical PBV in the trachea and nasal swabs of the same animal, with no amplification in the lung, may indicate an active replication and infection of the upper respiratory tract. Furthermore, deer N329 showed coinfection of GI and GII in both trachea and nasal swabs. Interestingly, each of the two GII trachea/nasal swabs testing positive showed identical sequences in the corresponding faecal specimen. The presence of respiratory viruses in faeces has previously been reported in humans [45,46] and wild ruminants [47]. Detection of PBV GI/II in the upper respiratory tract of deer in the present study, GI/II in respiratory tract swab specimens of pigs [14], GI in the respiratory tract of humans [12,13], GI in throat swab of monkeys [15] and GI/II in a nasopharyngeal swab of cattle [15], lead us to hypothesise whether these viruses might not only be potential enteric pathogens, but also respiratory pathogens. Detection of both GI and GII in deer respiratory tract samples highlights the need to expand our knowledge on picobirnavirus tropism.

A structured Bayesian coalescent model was used to infer PBV GII dynamics between the southern (Victoria) and northern (New South Wales) sampling locations. The maximum clade credibility Bayesian tree was broadly congruent with the tree from the maximum likelihood (ML) analysis. The Bayesian analysis pointed to the possible role of Victoria as a PBV GII distribution source for the southeast of Australia. Indeed, the analyses identified Victoria as the location of origin for this group of viruses with more than 80% confidence. Similarly, the PBV GII virus population size in Victoria would also appear to be larger. While these results provide a first insight into the PBV GII dynamics, we acknowledge that the accuracy of the population size parameters in our analysis was limited, as reflected by the wide HPD. The same applies to the estimation of the migration rates between subpopulations, in which case the uncertainty of the estimates was particularly high. This is not surprising as it is well known that it is difficult to accurately estimate these parameters (more so with non-informative priors as it was in our case) especially when the sample size is relatively small as it was in this study [48]. This is further complicated when migration rates are small. Therefore, we recommend that future studies expand the data presented here to improve these estimates.

To better understand the epidemiology of PBVs GII in deer and to define whether transmission to domestic animals occurs, there is a need to conduct a more intensive PBV surveillance in deer and cattle, possibly also including other geographical regions of Australia. However, whether GI and/or GII can also play a causal role in respiratory diseases remains to be determined. The lack of a suitable mammalian cell culture system and/or gnotobiotic animal models where PBVs are successfully propagated makes it difficult to determine the tissue tropism and pathogenesis of PBVs in mammals. This study illustrates how novel molecular techniques can provide a new understanding of viral epidemiology and evolution. Future studies will be necessary to understand the dynamics of virus circulation. Although much remains to be understood regarding the epidemiology of PBV, our study suggests that PBV may be widely distributed in deer and cattle in southeastern Australia. In conclusion, this first phylogenetic survey provides new knowledge and understanding of the putative host range and tropism of PBVs and confirms the high genetic diversity of GI.

## Figures and Tables

**Table 1:** Summary of sequences matching to PBV strains in barcoded libraries of deer samples.

Name	Source	Contig Name	Accession Number	Contig Length	Best Blast Hit	Accession Number	Query Cover	Identity
Library_D1	Plasma	Contig 1	MZ606752	1580 bp	*Picobirnavirus* sp.	QQM99858.1	95%	79%
Fallow deer ^a^		Contig 2	MZ606753	1507 bp	Bovine picobirnavirus	ATY68933.1	96%	87%
		Contig 3	MZ606754	1294 bp	Bovine picobirnavirus	ATY68936.1	95%	80%
		Contig 4	MZ606755	1180 bp	Bovine picobirnavirus	ATY68933.1	95%	80%
		Contig 5	MZ606756	891 bp	Bovine picobirnavirus	ATY68933.1	99%	82%
Library_D2	Serum	Contig 1	MZ606757	1719 bp	Bovine picobirnavirus	ATY68933.1	94%	78%
Rusa deer ^b^		Contig 2	MZ606758	1581 bp	Chicken picobirnavirus	AXL64616.1	97%	68%
		Contig 3	MZ606759	1463 bp	Bovine picobirnavirus	ATY68933.1	91%	82%
		Contig 4	MZ606760	1206 bp	Macaque picobirnavirus	AVD54063.1	96%	76%
		Contig 5	MZ606761	1092 bp	Bovine picobirnavirus	ATY68933.1	96%	83%
Library_D3	Serum	Contig 1	MZ606762	1693 bp	Fox picobirnavirus	AGK45545.1	92%	59%
Rusa deer ^b^		Contig 2	MZ606763	1256 bp	*Picobirnavirus* sp.	QQM99859.1	99%	76%
		Contig 3	MZ606764	1188 bp	Simian picobirnavirus	ATY68955.1	86%	81%
		Contig 4	MZ606765	899 bp	Marmot picobirnavirus	AVX53282.1	99%	70%
Library_D5	Serum	Contig 1	MZ606766	1689 bp	Marmot picobirnavirus	AVX53282.1	93%	67%
Rusa deer ^b^		Contig 2	MZ606767	1553 bp	Bovine picobirnavirus	ATY68941.1	94%	77%
		Contig 3	MZ606768	1547 bp	Macaque picobirnavirus	AVD54060.1	94%	72%
		Contig 4	MZ606769	1417 bp	Bovine picobirnavirus	ATY68939.1	96%	79%
		Contig 5	MZ606770	1400 bp	Marmot picobirnavirus	AVX53270.1	99%	82%
		Contig 6	MZ606771	1269 bp	*Picobirnavirus* sp.	YP009553306.1	97%	81%
		Contig 7	MZ606772	1219 bp	Bovine picobirnavirus	ATY68942.1	96%	72%
		Contig 8	MZ606773	960 bp	Marmot picobirnavirus	AVX29472.1	98%	72%
		Contig 9	MZ606774	902 bp	Bovine picobirnavirus	ATY68936.1	99%	86%
		Contig 10	MZ606775	897 bp	Macaque picobirnavirus	AVD54061.1	97%	82%
		Contig 11	MZ606776	888 bp	*Picobirnavirus* sp.	AVD97044.1	98%	69%

^a^*Dama dama*, ^b^*Rusa timorensis*.

**Table 2:** Diagnostic test results for PBV detection in deer.

Variable	Category	Total Subjects	PBV-Positive (%)	PBV Genogroup (*n*)		
				GI	GII	GI and GII
Species	Fallow deer ^a^	60	32 (53.3)	27	12	7
	Sambar deer ^b^	11	4 (36.4)	3	2	1
	Total	71	36 (50.7)	30	14	8
Location	Kiah (NSW)	48	23 (47.9)	19	11	7
	Yellingbo (VIC)	8	5 (62.5)	5	1	0
	Outer Melbourne (VIC)	5	2 (40)	1	1	0
	Bunyip (VIC)	10	6 (60)	5	1	0
Sample type	Serum	71	12 (16.9)	7	4	0
	Spleen	33	0	0	0	0
	Faeces	71	27 (38.1)	23	9	5
	Trachea	15	3 (20)	2	2	1
	Lung	15	0	0	0	0
	Nasal swabs	15	7 (46.7)	6	4	3

^a^*Dama dama*, ^b^*Rusa unicolor* PBV = picobirnavirus, GI = genogroup I, GII = genogroup II, NSW = New South Wales, VIC = Victoria.

**Table 3:** Diagnostic test results for PBV detection in cattle.

Location	Total Subjects	PBV-Positive (%)	PBV Genogroup (*n*)		
			GI	GII	GI and GII
Kiah (NSW)	8	6 (75)	5	2	1
Outer Melbourne (VIC)	4	3 (75)	3	1	1
Bunyip State Park (VIC)	11	10 (90.9)	10	5	5
Total	23	19 (82.6)	18	8	7

PBV = picobirnavirus, GI = genogroup I, GII = genogroup II, NSW = New South Wales, VIC = Victoria.

**Table 4:** Multi-specimen detection of PBV genogroup I.

Deer Code	Location	Deer Species	PBV GI Specimen Result			
			Faeces	Serum	Trachea	Nasal Swab
N308	Kiah (NSW)	Fallow	+	+	NS	NS
N311	Kiah (NSW)	Fallow	+	+	NS	NS
N321	Kiah (NSW)	Fallow	+	−	−	+
N325	Kiah (NSW)	Fallow	+	−	−	+
N326	Kiah (NSW)	Fallow	+	+	−	−
N327	Kiah (NSW)	Fallow	−	−	+	+
N329	Kiah (NSW)	Fallow	−	+	+	+

+ = positive, − = negative, NS = no sample.

**Table 5:** Multi-specimen detection of PBV genogroup II.

Deer Code	Location	Deer Species	PBV GII Specimen Result			
			Faeces	Serum	Trachea	Nasal Swab
N325	Kiah (NSW)	Fallow	+	−	−	+
N329	Kiah (NSW)	Fallow	+	−	+	+
N331	Kiah (NSW)	Fallow	+	−	+	+

+ = positive, − = negative, NS = no sample.

## Data Availability

All data can be obtained from the authors on request.

## Supplementary Materials

The following are available online at https://www.mdpi.com/article/10.3390/v13081492/s1, Figure S1: Alignment of the partial RNA-dependent RNA polymerase (RdRp) gene sequences of PBVs GII discovered in the present study.
