# Supplementary material for: Molecular Epidemiology and Characterization of Picobirnavirus in Wild Deer and Cattle from Australia: Evidence of Genogroup I and II in the Upper Respiratory Tract
Source: Viruses. 2021 Jul 29;13(8):1492. doi: 10.3390/v13081492 (PMC8402760; doi:10.3390/v13081492)
Supplement: Supplementary file 1 [file viruses-13-01492-s001.zip › viruses-1283601-supplementary.pdf]

```
             ....|....| ....|....| ....|....| ....|....| ....|....| ....|....| ....|....| ....|....| ....|....| ....|....|
                 5          15         25         35         45         55         65         75         85         95
LN7_f        *******AGC T**CT*AA*C **GTTTACG* *TG*G*AAGT AATT*AGAAA GAAT*ACAGA AATCT*CAGC GCAGTGGG*A CGA**C***C TAT*G**T**

N329_f       *******GTT A**TT*AG*C **ATTTTCC* *CT*A*AAAG TAAT*CAGAT GCTT*ACAGC ATTCA*CAAC TCCTTGGA*T AGA**T***C TAT*A**G**
N329_ns      *******GTT A**TT*AG*C **ATTTTCC* *CT*A*AAAG TAAT*CAGAT GCTT*ACAGC ATTCA*CAAC TCCTTGGA*T AGA**T***C TAT*A**G**
N329_t       *******GTT A**TT*AG*C **ATTTTCC* *CT*A*AAAG TAAT*CAGAT GCTT*ACAGC ATTCA*CAAC TCCTTGGA*T AGA**T***C TAT*A**G**
N360_s       *******GTT A**TT*AG*C **ATTTTCC* *CT*A*AAAG TAAT*CAGAT GCTT*ACAGC ATTCA*CAAC TCCTTGGA*T AGA**T***C TAT*A**G**
N340_s       *******GTT A**TT*AG*C **ATTTTCC* *CT*A*AAAG TAAT*CAGAT GCTT*ACAGC ATTCA*CAAC TCCTTGGA*C AGA**T***T TAT*G**G**
N332_ns      *******GTT A**TT*AG*C **ATTTTCC* *CT*A*AAAG TAAT*CAGAT GCTC*CCAGC ATTCG*CTAC TCCTTGGG*T CGT**T***C TAT*G**G**

N346_s       *******TAT T**CT*AG*T **GTTTACC* *CT*G*AAGT GATC*AGGAT GCTC*TGTTT CTTCA*AAGC TAAGTGGA*A AGA**T***C TAT*A**G**

LV15_f       *******AGT A**CT*AA*A **ATTTTCC* *CG*G*AAAT CGTC*AGGAG TCGC*TCAAC ACTCG*CAAG TAGAGAAG*G ACT**C***C TAT*A**G**
V85_s        *******AGT A**CT*AA*A **ATTTTCC* *CG*G*AAAT CGTC*AGGAG TCGC*TCAAC ACTCG*CAAG TAGAGAAG*G ACT**C***C TAT*A**G**
N320_f       *******AGT A**CT*AA*A **ATTTTCC* *CG*G*AAAT CGTC*AGGAG TCGC*TCAAC ACTCG*CAAG TAGAGAAG*G ACT**C***T TAT*A**G**
N327_f       *******AGT A**CT*AA*A **ATTTTCC* *CG*G*AAAT CGTC*AGGAG TCGC*TCAAC ACTCG*CAAG TAGAGAAG*G ACT**C***T TAT*A**G**
N325_f       *******AGT A**CT*AA*A **ATTTTCC* *CG*G*AAAT CGTC*AGGAG TCGC*TCAAC ACTCG*CAAG TAGAGAAG*G ACT**C***T TAT*A**G**
N331_f       *******AGT A**CT*AA*A **ATTTTCC* *CG*G*AAAT CGTC*AGGAG TCGC*TCAAC ACTCG*CAAG TAGAGAAG*G ACT**C***T TAT*A**G**
N331_n       *******AGT A**CT*AA*A **ATTTTCC* *CG*G*AAAT CGTC*AGGAG TCGC*TCAAC ACTCG*CAAG TAGAGAAG*G ACT**C***T TAT*A**G**
N331_t       *******AGT A**CT*AA*A **ATTTTCC* *CG*G*AAAT CGTC*AGGAG TCGC*TCAAC ACTCG*CAAG TAGAGAAG*G ACT**C***T TAT*A**G**
N349_f       *******AGT A**CT*AA*A **ATTTTCC* *CG*G*AAAT CGTC*AGGAG TCGC*TCAAC ACTCG*CAAG TAGAGAAG*G ACT**C***T TAT*A**G**

LV2_f        *******GAC A**CC*GG*A **AGCTGGC* *TG*C*TTG- AATC*AGAGT TGGA*CACAC ATTCG*CACT TCAGTGGG*A AGA**C***T GCA*T**G**
LV7_f        *******GAC A**CC*GG*A **AGCTGGC* *TG*C*TTG- AATT*AGAAT TGTG*CGCTC AGTCC*CGCT TGAGTGGG*T AGA**C***T GCA*G**A**
LV10_f       *******GAC A**CC*GG*A **AGCTGGC* *TG*C*TTG- AATT*AGAAT TGTG*CGCTC AGTCC*CGCT TGAGTGGG*T AGA**C***T GCA*G**A**
LV12_f       *******GAC A**CC*GG*A **AGCTGGC* *TG*C*TTG- AATT*AGAAT TGTG*CGCTC AGTCC*CGCT TGAGTGGG*T AGA**C***T GCA*G**A**
V92_f        *******GAC A**CC*GG*A **AGCTGGC* *TG*C*TTG- AATT*AGAAT TGTG*CGCTC AGTCC*CACT TGAGTGGG*T AGA**C***T GCA*G**A**
N325_ns      *******GAC A**CC*AA*A **AGCCGGT* *CG*C*TTA- AATC*AGGAT TGTA*TGCTC ATAGC*CACT CGAGTGGG*T CGA**C***T GCA*G**A**
             CGATGTC     AA  T  T  GA       T T  C C         C          T           C             T     GA TAC     C CC TG


             ....|....| ....|....| ....|....| ....|....| ....|....| ....|....| ....|....| ....|....| ....|....| ...
                105        115        125        135        145        155        165        175        185
```

```
LN7_f        *CTT**A*TT **GGAC**T* *GCTTACCC* C**CAAG*** ***---AGTG GCGAACCAA* C*AT****** ********** *G******** ***

N329_f       *CAA**C*TT **ATTC**A* *AGAAACAC* G**TAAG*** ***---CAAG GCCAGCATA* T*AT****** ********** *A******** ***
N329_ns      *CAA**C*TT **ATTC**A* *AGAAACAC* G**TAAG*** ***---CAAG GCCAGCATA* T*AT****** ********** *A******** ***
N329_t       *CAA**C*TT **ATTC**A* *AGAAACAC* G**TAAG*** ***---CAAG GCCAGCATA* T*AT****** ********** *A******** ***
N360_s       *CAA**C*TT **ATTC**A* *AGAAACAC* G**TAAG*** ***---CGAG GCCAGCATA* T*AT****** ********** *A******** ***
N340_s       *CAA**C*TT **ATTC**A* *AGAAACAC* G**TAAG*** ***---AAAG GCCAGCATA* T*AT****** ********** *A******** ***
N332_ns      *TTG**C*TT **ATTC**A* *AGAAACGC* G**CAAG*** ***---AAAG GCCAACATA* T*TA****** ********** *A******** ***

N346_s       *CAA**C*AT **AGAT**T* *GAGGACTT* A**TCTT*** ***---ACGG GCCAAAACG* A*AT****** ********** *G******** ***

LV15_f       GGAG**G*AT **GGTC**T* *GCGTACTC* A**CTCT*** ***CCTAACG GCTCACAGG* T*TT****** ********** *G******** ***
V85_s        GGAG**G*AT **GGTC**T* *GCGTACTC* A**CTCT*** ***CCTAACG GCTCACAGG* T*TT****** ********** *G******** ***
N320_f       GGAG**G*AT **GGTC**T* *GCGTACTC* A**CTCT*** ***CCTAACG GCTCACAGG* T*TT****** ********** *G******** ***
N327_f       GGAG**G*AT **GGTC**T* *GCGTACTC* A**CTCT*** ***CCTAACG GCTCACAGG* T*TT****** ********** *G******** ***
N325_f       GGAG**G*AT **GGTC**T* *GCGTACTC* A**CTCT*** ***CCCAACG GCTCACAGG* T*TT****** ********** *G******** ***
N331_f       GGAG**G*AT **GGTC**T* *GCGTACTC* A**CTCT*** ***CCCAACG GCTCACAGG* T*TT****** ********** *G******** ***
N331_n       GGAG**G*AT **GGTC**T* *GCGTACTC* A**CTCT*** ***CCCAACG GCTCACAGG* T*TT****** ********** *G******** ***
N331_t       GGAG**G*AT **GGTC**T* *GCGTACTC* A**CTCT*** ***CCCAACG GCTCACAGG* T*TT****** ********** *G******** ***
N349_f       GGAG**G*AT **GGTC**T* *GCGCACTC* A**CTCT*** ***CCTAACG GCTCACAGG* T*TT****** ********** *G******** ***

LV2_f        *CTT**T*TT **GCAC**A* *AGGTGTGC* T**GAAG*** ***CCGATGA ACGCTGTGA* C*CT****** ********** *G******** ***
LV7_f        *CTC**A*TC **GCAT**T* *AAGTGTGC* A**AAAG*** ***CCCGACA AAGCAGCGA* A*TA****** ********** *G******** ***
LV10_f       *CTC**A*TC **GCAT**T* *AAGTGTGC* A**AAAG*** ***CCCGACA AAGCAGCGA* A*TA****** ********** *G******** ***
LV12_f       *CTC**A*TC **GCAT**T* *AAGTGTGC* A**AAAG*** ***CCCGACA AAGCAGCGA* A*TA****** ********** *G******** ***
V92_f        *CTC**A*TC **GCAT**C* *AAGTGTGC* G**AAAG*** ***CCCGACA AAGCAGCGA* C*TT****** ********** *G******** ***
N325_ns      *TTG**T*TT **CCAT**G* *GAGTGTGC* G**AAAG*** ***CCTCATG AAGCAGCAA* A*TT****** ********** *G******** ***
             G   GG T   GA    GT A A        T  AC    CAG TGG                 T  G  GGTGGA GACACCACTA A ATGGATGC ACA
```

Figure S1. Alignment of the partial RNA-dependent RNA polymerase (RdRp) gene sequences of PBVs GII discovered in the present study. A '*'denotes an identical nucleotide residue, whilst '-' indicates absence of nucleotide residue. Numbers to the top indicate the positions of the nucleotide for respective PBV strains. Gap positions are highlighted in green.
